# Effectiveness of Low-Level Red Light in Controlling Myopia Progression in Children: A Systematic Review and Meta-Analysis

**DOI:** 10.7759/cureus.80336

**Published:** 2025-03-10

**Authors:** Yazeed B Alaql, Saleh A Alkhalaf, Bader A Almuqaythil, Areen E Almatham, Sulaiman K Alherbish, Lama Alharbi, Ziyad A Alsweed, Faisal A Al-Harbi, Meshari Y Aloufi, Dora H AlHarkan

**Affiliations:** 1 Ophthalmology, Qassim University, Buraydah, SAU; 2 Ophthalmology Department, Medical College, Qassim University, Qassim, SAU

**Keywords:** axial length (al), childhood myopia, low-level red light (llrl), spherical equivalent refraction (ser), subfoveal choroidal thickness (sfct)

## Abstract

This study aimed to evaluate the effectiveness of low-level red light (LLRL) therapy compared to single-vision spectacles (SVS) in controlling myopia progression, focusing on spherical equivalent refraction (SER), axial length (AL), and subfoveal choroidal thickness (SFCT). A comprehensive search was conducted across PubMed, Cochrane Library, ScienceDirect, and Web of Science, including only randomized controlled trials (RCTs) involving myopic patients aged 16 years or younger. Mean differences were calculated with 95% CIs, and the I² statistic was used to assess heterogeneity among studies. The meta-analysis included 11 RCTs. At three months, significant differences were observed between LLRL and SVS in SER (estimate: 0.225; 95% CI: 0.042, 0.408; p = 0.016) and AL (estimate: -0.137; 95% CI: -0.280, 0.007; p = 0.062). At six months, significant differences were noted in SER (estimate: 0.450; 95% CI: 0.351, 0.550; p < 0.001), AL (estimate: -0.179; 95% CI: -0.278, -0.079; p < 0.001), and SFCT (estimate: 35.281; 95% CI: 23.230, 47.331; p < 0.001). By 12 months, the differences remained significant for SER (estimate: 0.806; 95% CI: 0.675, 0.937; p < 0.001) and AL (estimate: -0.294; 95% CI: -0.416, -0.172; p < 0.001). These findings suggest that LLRL therapy effectively reduces myopia progression in children, as indicated by improvements in SER, slower AL growth, and stabilization or slight increases in SFCT. However, further studies are necessary to determine the optimal dosage, long-term safety, and overall efficacy of LLRL therapy.

## Introduction and background

Myopia, commonly known as “near-sightedness,” is a prevalent refractive error that impairs the ability to see distant objects clearly [1,2]. This condition is particularly common among children and young adults, with its prevalence rising significantly after the COVID-19 pandemic, likely due to reduced outdoor activities and increased screen time [1]. An extensive analysis of 145 studies estimated the global prevalence of myopia and high myopia in 2010 to be approximately 28% and 4%, respectively. Moreover, projections suggest that by 2050, nearly half of the world’s population - about 49.8% - will be affected by myopia [3].

Managing myopia is particularly challenging in today’s screen-centered lifestyle. Over the years, several treatment options have demonstrated effectiveness in controlling myopia progression, including pharmacological interventions like atropine eye drops and specialized lenses such as multifocal lenses, peripheral defocus contact lenses, and orthokeratology [1].

Although these treatments have shown efficacy, each comes with potential side effects that may limit their overall benefits [4]. For instance, orthokeratology carries a heightened risk of microbial keratitis, a severe eye infection that can lead to blindness [5]. Meanwhile, atropine eye drops can cause photophobia and other adverse effects [6]. Given these limitations, there is an urgent need to develop newer, safer, and more effective strategies to manage and slow the progression of myopia in children.

Previous research has indicated that lifestyle modifications, such as reducing screen time and increasing outdoor activities, can significantly lower the risk of developing myopia in individuals without underlying health conditions. However, implementing outdoor programs is often suboptimal in various settings, making it an inefficient large-scale prevention strategy. This highlights the potential value of developing and optimizing light therapy devices as a more controlled and reliable approach [7].

Low-level red light (LLRL) therapy is a noninvasive, painless technique that stimulates tissue healing and reduces inflammation, with proven effectiveness in managing various medical conditions [4]. Recent randomized controlled trials (RCTs) have investigated the efficacy of repeated LLRL (RLRL) therapy in slowing myopia progression in children compared to controls, showing significant reductions in myopia progression without any reported treatment-related side effects [8,9].

This systematic review and meta-analysis aimed to assess the effectiveness of combining RLRL therapy with single-vision spectacles (SVS) in slowing myopia progression in the pediatric population. Additionally, it aimed to evaluate how RLRL therapy influences subfoveal choroidal thickness (SFCT), spherical equivalent refraction (SER), and axial length (AL) at various follow-up periods.

## Review

Materials and methods

This study was conducted following the Preferred Reporting Items for Systematic reviews and Meta-Analyses (PRISMA) 2020 guidelines [10] and was registered in PROSPERO under the accession number CRD42024591478.

Literature Search

A comprehensive search of PubMed, Cochrane Library, ScienceDirect, and Web of Science databases was performed, covering publications from database inception up to September 2024. The search strategy included the terms: (“Myopia” OR “Myopias” OR “Nearsightedness” OR “Nearsightednesses”) AND (“Low-Level Red Light” OR “Repeated Low-Level Red Light” OR “RLRL”). Additionally, references from the selected studies were examined to identify relevant studies not captured in the initial search.

Study Selection

The initial search results were imported into Rayyan software (www.rayyan.ai) for a preliminary review of titles and abstracts by two independent authors who assessed their eligibility [11]. Full-text screening was subsequently conducted for studies meeting the inclusion criteria. Any disagreements were resolved through discussion with an additional author.

Inclusion Criteria

Studies were eligible if they were RCTs published in English, comparing LLRL therapy with SVS in children with myopia aged 16 years or younger. Studies had to report at least one of the following outcomes: SER, SFCT, or AL, with a minimum follow-up duration of three months.

Exclusion Criteria

Studies were excluded if they were non-English publications, nonhuman studies, non-RCT designs, involved patients with ocular comorbidities, or included participants with prior use of other myopia control methods.

Data Extraction

Two authors independently extracted data using a standardized Google Sheet form (Google LLC, Mountain View, CA, USA). The collected information included study design, location and number of centers, total number of participants, inclusion criteria, LLRL description, follow-up period, study conclusions, patient numbers in each arm, age, sex, AL, SER, and SFCT. A third author reviewed the final dataset for accuracy.

Risk of Bias Assessment

The quality of the included RCTs was assessed using the revised Cochrane Risk-of-Bias tool for Randomized Trials (RoB 2) [12]. This tool evaluates studies across five domains focusing on trial design, execution, and reporting. Each domain includes “signaling questions” aimed at identifying potential biases. Based on the responses, studies were classified as having “low risk” (all domains have a low risk of bias), “some concerns” (at least one domain has some concerns), or “high risk” (at least one domain has a high risk of bias or multiple domains raise concerns). The assessment was performed independently by two authors, and discrepancies were resolved through consensus with a third author.

Data Synthesis and Analysis

Statistical analysis was performed using Review Manager (RevMan 5.4). For continuous variables - SER, SFCT, and AL - mean differences (MDs) were calculated with 95% CIs. Effect estimates were derived from differences between the LLRL and control groups across various follow-up periods. A random-effects model was applied to account for variability among studies.

Heterogeneity was assessed using the I² statistic, where values exceeding 50% indicated moderate to high heterogeneity. Cochran’s Q test was used to evaluate statistical significance, with a p-value below 0.10 suggesting significant heterogeneity. Subgroup analyses were conducted based on factors such as LLRL power settings and follow-up duration to identify potential sources of variability. Results were reported as MDs with corresponding 95% CIs, and a p-value of less than 0.05 was considered statistically significant.

Results

Results of the Primary Literature Search

**Figure 1:**
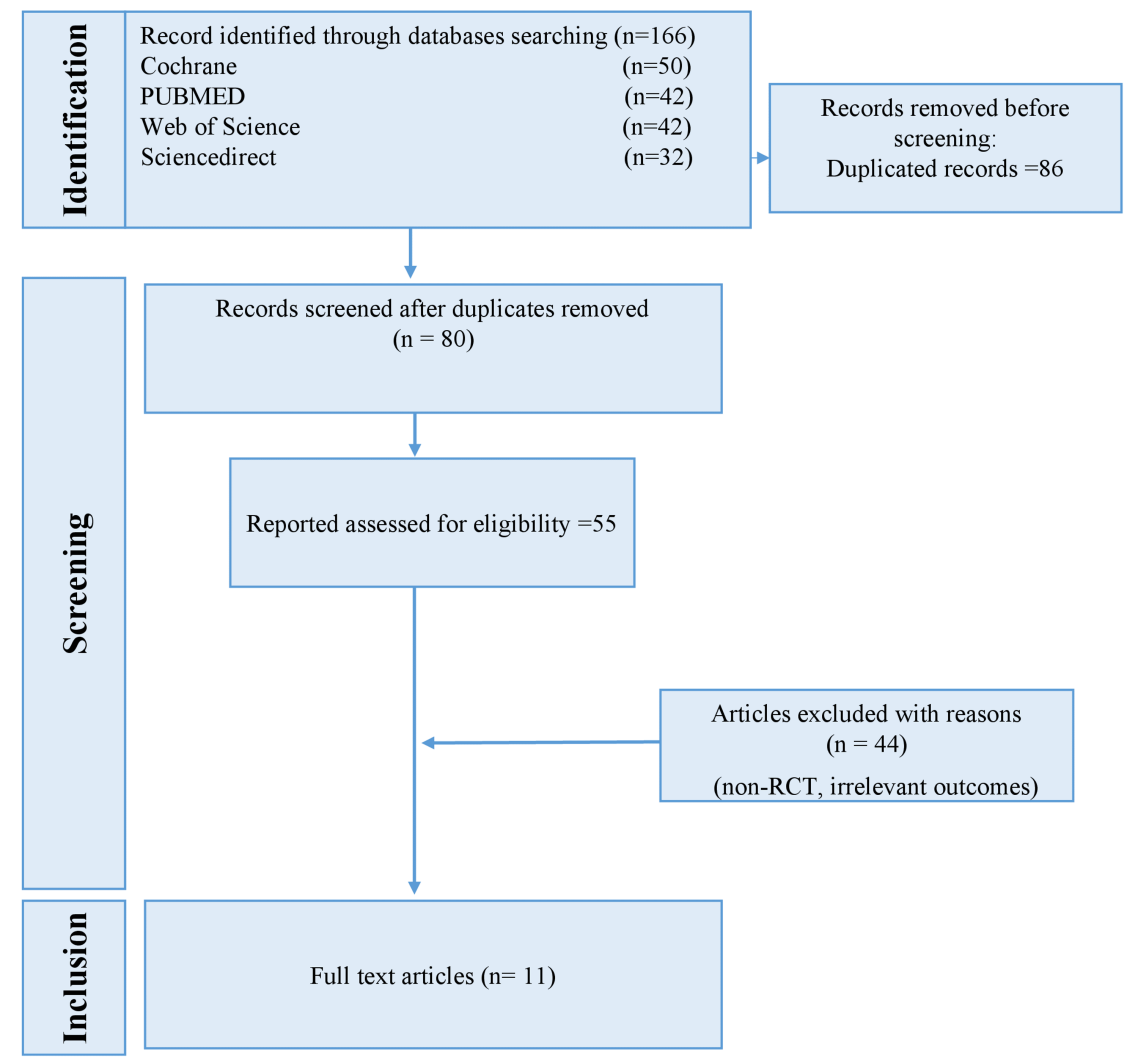
PRISMA flow diagram illustrating the study selection process for the systematic review and meta-analysis PRISMA, Preferred Reporting Items for Systematic reviews and Meta-Analyses; RCT, randomized controlled trial

The literature search process involved a systematic screening and evaluation of relevant studies. A total of 166 articles were identified through searches in the PubMed (n = 42), Cochrane Library (n = 50), Web of Science (n = 42), and ScienceDirect (n = 32) databases. After the removal of duplicates, 80 unique studies remained. Titles and abstracts of these studies were screened, resulting in 55 studies selected for a detailed full-text review. Following the application of inclusion and exclusion criteria, 11 studies were deemed eligible and included in this systematic review and meta-analysis (Figure 1).

Characteristics of the Included Studies

The included studies collectively evaluated the efficacy of LLRL therapy in controlling myopia progression among children and adolescents. Most of the research was conducted in China and comprised RCTs with sample sizes ranging from 73 to 336 participants. The typical inclusion criteria involved participants aged six to 16 years with varying levels of myopia, usually defined by an SER ranging from -6.00 D to -0.50 D, and astigmatism limits between 1.5 D and 3.0 D. The LLRL interventions mainly utilized red light wavelengths around 650 nm, administered for approximately three minutes twice daily. Follow-up durations varied across studies, ranging from three to 12 months. Many studies concluded that LLRL therapy effectively slowed myopia progression without causing significant short-term side effects. Interestingly, higher power settings of up to 1.20 mW appeared to be more effective in some instances. Overall, LLRL therapy emerged as a promising, noninvasive, and safe approach for managing myopia in children (Table 1).

**Table 1:** Summary of previous studies characteristics AL, axial length; RCT, randomized control trial; RLRL, repeated low-level red light; SER, spherical equivalent refraction

Author and year	Study design	Country	Centers	Participants	Inclusion criteria	RLRL description	Follow-up period	Study conclusion
Dong et al. (2023) [8]	Double-blind RCT	China	Two centers	112	Age: 7-12; SER ≥ -0.50D; Astigmatism: ≤1.5D	650 nm, 0.29 mW, twice daily for three minutes	Six months	RLRL significantly reduced myopia progression.
Jiang et al. (2022) [9]	Parallel-group, single-blind RCT	China	Five centers	264	Age: 8-13; SER: -1.00 to -5.00D; Astigmatism: ≤2.5D	650 nm, 0.29 mW, twice daily for three minutes	One, three, six, and 12 months	RLRL is a promising alternative for myopia control in children.
Xiong et al. (2024) [13]	RCT	China	Single center	73	Age: 6-14; SER: -6.00D to -0.50D; Astigmatism: ≤3.0D	650 nm, 0.9 mW, twice daily for three minutes	One, three, and six months	RLRL effectively controls myopia progression within six months.
Xu et al. (2024) [14]	Parallel-group, single-blind RCT	China	Six centers	192	Age: 6-16; SER: ≥ -4.0D; Astigmatism: ≤2.0D	650 nm, 0.29 mW, twice daily for three minutes	One, three, six, nine, and 12 months	Effective for high myopia control, with 53.3% showing axial shortening
Zhu et al. (2024) [15]	RCT	China	Single center	115	Age: 6-14; SER: -0.50 to -6.00D	650 nm ± 10 nm, 0.29 mW, twice daily for three minutes	Three, six, and 12 months	Safe and effective treatment for slowing myopia progression
Cao et al. (2024) [16]	RCT	China	Single center	336	Age: 6-12; SER: -6D to 3D; Astigmatism: ≤2.5D	650 nm, twice daily for three minutes	Six and 12 months	RLRL effectively slows SER and AL progression without safety concerns.
Liu et al. (2024) [17]	Parallel-group RCT	China	Single center	170	Age: 8-13; SER: -6.00D to -1.00D; Astigmatism: ≤1.50D	650 nm, twice daily for three minutes	One, three, six, nine, and 12 months	RLRL delays myopia progression and reduces incidence in pre-myopic children.
Zhou et al. (2024) [18]	Parallel-group, single-masked RCT	China	Single center	200	Age: 6-15; SER: ≥ -0.50D; Astigmatism: ≤2.5D	650 nm, 0.37, 0.60, 1.20 mW, twice daily for three minutes	One, three, and six months	Higher power RLRL may enhance effectiveness in controlling myopia.
Chen et al. (2023) [19]	RCT	China	Single center	102	Age: 6-13; SER: -0.75 to -6.00D; Astigmatism: ≤1.5D	635 nm, 0.35 mW, twice daily for three minutes	Three, six, nine, and 12 months	RLRL may improve accommodative function and prevent myopia progression.
Tian et al. (2022) [20]	Single-masked RCT	China	Single center	224	Age: 6-12; SER: -6D to -0.5D; Astigmatism: ≤2.5D	650 nm, twice daily for three minutes	Six months	650 nm RLRL effectively slows myopia progression without short-term side effects.
Xiong et al. (2021) [21]	RCT	China	Single center	229	Age: 6-16; SER: ≤ -0.50D	650 nm, 2 ± 0.5 mW, twice daily for three minutes	One, three, and six months	RLRL effectively slows myopia progression.

When comparing the control and LLRL treatment groups, participants were generally similar in age and sex distribution, with most studies involving children between the ages of 6 and 16. The average age of participants typically ranged from nine to 11 years, with a nearly equal male-to-female ratio. Baseline SER and AL values showed slight variations between the control and LLRL groups, but these differences were generally minimal. Across both groups, SER values ranged from approximately -2 D to -5 D, while AL measurements varied between 24 mm and 26 mm. In studies where SFCT measurements were reported, LLRL treatment appeared to help maintain or slightly increase SFCT values compared to control groups. Overall, LLRL treatment was associated with a more significant reduction in myopia progression than controls, highlighting its potential as an effective intervention for managing childhood myopia (Table 2).

**Table 2:** Differences between control and LLRL groups at baseline characteristics AL, axial length; LLRL, low-level red light; SER, spherical equivalent refraction; SFCT, sub-foveal choroidal thickness

Author and year	Control group							LLRL group								
	No. of patients	Age range	Age (mean ± SD)	Male (N)	Female (N)	SER (D)	AL (mm)	SFCT (µm)	No. of patients	Age range	Age (mean ± SD)	Male (N)	Female (N)	SER (D)	AL (mm)	SFCT (µm)
Dong et al. (2023) [8]	55	7-12	9.86 ± 1.41	30	26	-2.82 ± 1.86	24.6 ± 0.96	NA	56	7-12	10.3 ± 2.07	26	30	-3.13 ± 1.91	24.7 ± 1.04	NA
Jiang et al. (2022) [9]	145	8-13	10.53 ± 3.66	73	72	-2.67 ± 1.06	24.62 ± 0.86	NA	119	8-13	10.46 ± 3.75	57	62	-2.49 ± 0.92	24.54 ± 0.67	NA
Xiong et al. (2024) [13]	37	6-14	9.00 ± 2.00	22	15	-2.22 ± 0.72	24.47 ± 0.58	274.76 ± 63.79	36	6-14	8.83 ± 2.06	17	19	-2.47 ± 1.39	24.38 ± 0.87	251.83 ± 65.27
Xu et al. (2024) [14]	95	6-16	11.20 (2.1)	44	51	-5.75 (1.17)	25.72 (0.87)	NA	97	6-16	10.40 (2.4)	63	34	-5.88 (1.69)	25.93 (1.03)	NA
Zhu et al. (2024) [15]	60	6-14	8.47 ± 2.10	31	24	-2.85 ± 1.71	24.40 ± 1.02	NA	55	6-14	8.96 ± 2.19	30	23	-3.02 ± 1.80	24.66 ± 0.93	NA
Cao et al. (2024) [16]	168	6-12	9.0 (1.9)	82	86	-2.1 (1.2)	24.2 (0.8)	299.3 (68.2)	168	6-12	9.1 (2.0)	78	90	-2.2 (1.2)	24.3 (0.9)	291.4 (71.6)
Liu et al. (2024) [17]	36	8-13	9.55 ± 1.13	17	19	-2.61 ± 0.98	24.58 ± 0.64	NA	32	8-13	9.37 ± 1.69	16	16	-2.91 ± 1.27	24.71 ± 0.92	NA
Zhou et al. (2024) [18]	50	6-15	9.03 ± 1.63	29	21	-2.10 ± 0.90	24.41 ± 0.90	268.74 ± 58.62	150	6-15	0.37 mW: 8.58 ± 1.46; 0.60 mW: 8.82 ± 1.45; 1.20 mW: 8.78 ± 1.49	0.37 mW: 30; 0.60 mW: 25; 1.20 mW: 26	0.37 mW: 20; 0.60 mW: 25; 1.20 mW: 24	0.37 mW: 1.72 ± 0.91; 0.60 mW: 2.01 ± 0.87; 1.20 mW: 2.08 ± 1.33	0.37 mW: 24.19 ± 0.79; 0.60 mW: 24.12 ± 0.88; 1.20 mW: 24.45 ± 0.91	0.37 mW: 251.54 ± 55.64; 0.60 mW: 244.80 ± 45.12; 1.20 mW: 252.06 ± 63.38
Chen et al. (2023) [19]	40	6-13	8.98 ± 1.92	25	15	-2.29 ± 0.77	24.57 ± 0.76	273.08 ± 54.37	46	6-13	9.00 ± 1.90	27	19	-2.54 ± 1.04	24.62 ± 0.97	259.00 ± 51.46
Tian et al. (2022) [20]	112	6-12	9.47 ± 1.59	57	55	-2.00 ± 1.13	24.20 ± 0.85	297.33 ± 81.11	112	6-12	9.66 ± 1.65	55	57	-2.17 ± 1.50	24.31 ± 0.92	295.00 ± 82.98
Xiong et al. (2021) [21]	74	7-14	10.33 ± 2.03	40	34	-3.32 ± 1.36	25.07 ± 0.87	286.81 ± 63.67	74	7-15	10.22 ± 2.38	40	34	-3.39 ± 2.17	25.07 ± 1.15	288.61 ± 59.59

The data presented in Table 2 compare the control and LLRL groups regarding SER, AL, and SFCT over time. In the control group, SER values progressively decreased from -0.87 at one month to -0.76 at 12 months, indicating continued myopic progression. Conversely, the LLRL group showed less myopic progression, with SER improving from -0.812 at one month to 0.055 at 12 months. Both groups experienced a gradual reduction in AL; the control group’s AL decreased from 5.03 at one month to 0.372 at 12 months, while the LLRL group showed a similar trend, with AL declining from 4.968 at one month to 0.0286 at 12 months. Regarding SFCT, the control group displayed a decline from 286.45 μm at one month to -22.26 μm at 12 months. In contrast, the LLRL group exhibited an increase from 311.84 μm at one month to 16.46 μm at 12 months. These findings suggest that the LLRL group experienced reduced myopic progression and better preservation of choroidal thickness over time compared to the control group (Table 3).

**Table 3:** Pooled outcomes among control and LLRL groups at different follow-ups AL, axial length; LLRL, low-level red light; SER, spherical equivalent refraction; SFCT, sub-foveal choroidal thickness

Parameter	Group	One month	Three months	Six months	Nine months	12 months
		No. of patients	Mean	No. of patients	Mean	No. of patients
SER	Control	290	-0.87	383	-0.73	777
	LLRL	270	-0.812	371	-0.492	775
AL	Control	382	5.03	475	3.67	777
	LLRL	366	4.968	467	3.52	775
SFCT	Control	74	286.45	114	137.205	289
	LLRL	74	311.84	120	172.305	290

Risk of Bias

**Figure 2:**
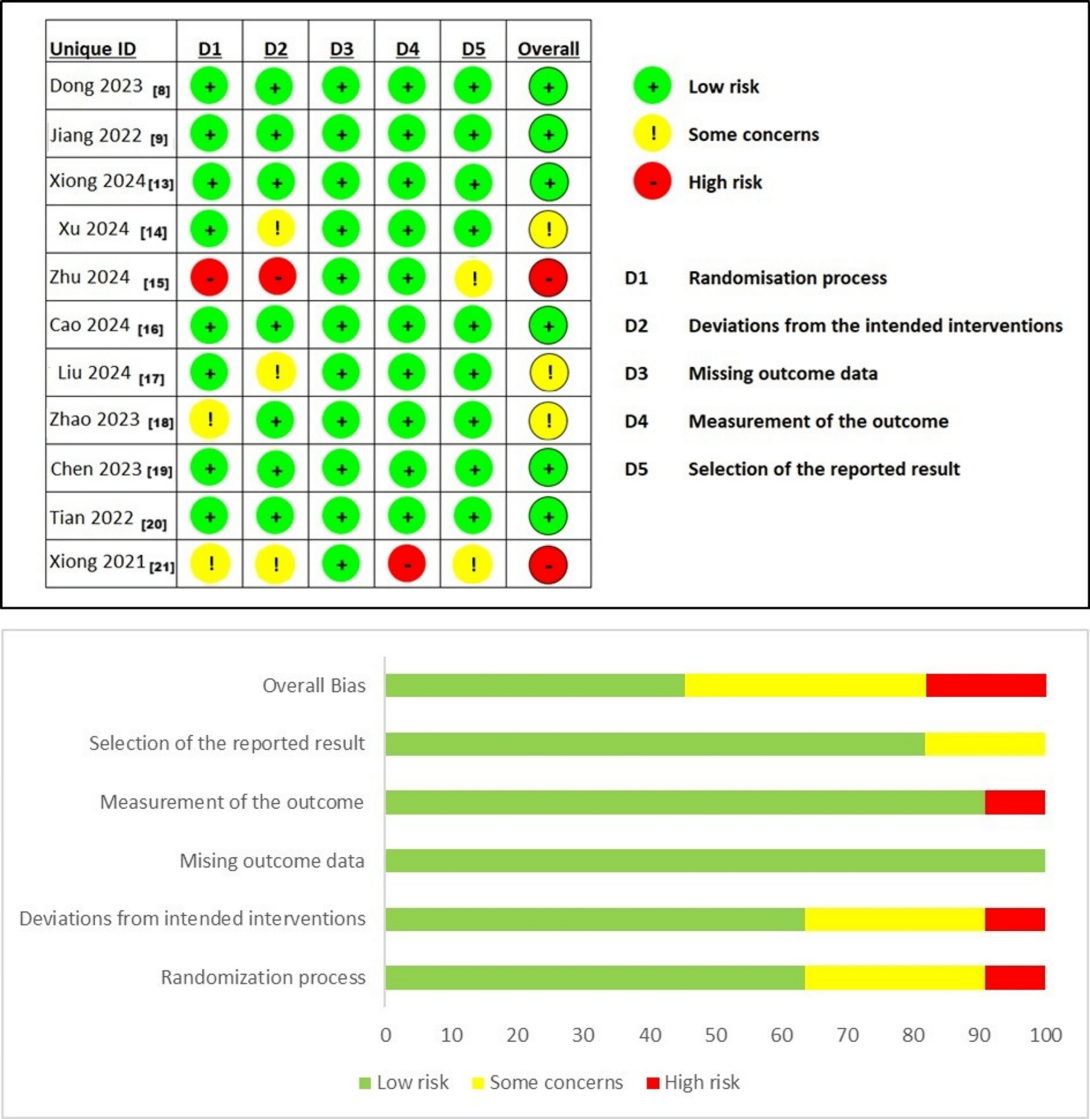
Risk of bias graph and summary [8,9,13-21]

The risk of bias in the included RCTs was evaluated using the RoB2 tool. Among the studies, six were deemed to have a low risk of bias, two were identified as having a high risk, and three were classified as raising some concerns (Figure 2).

Meta-Analysis

**Figure 3:**
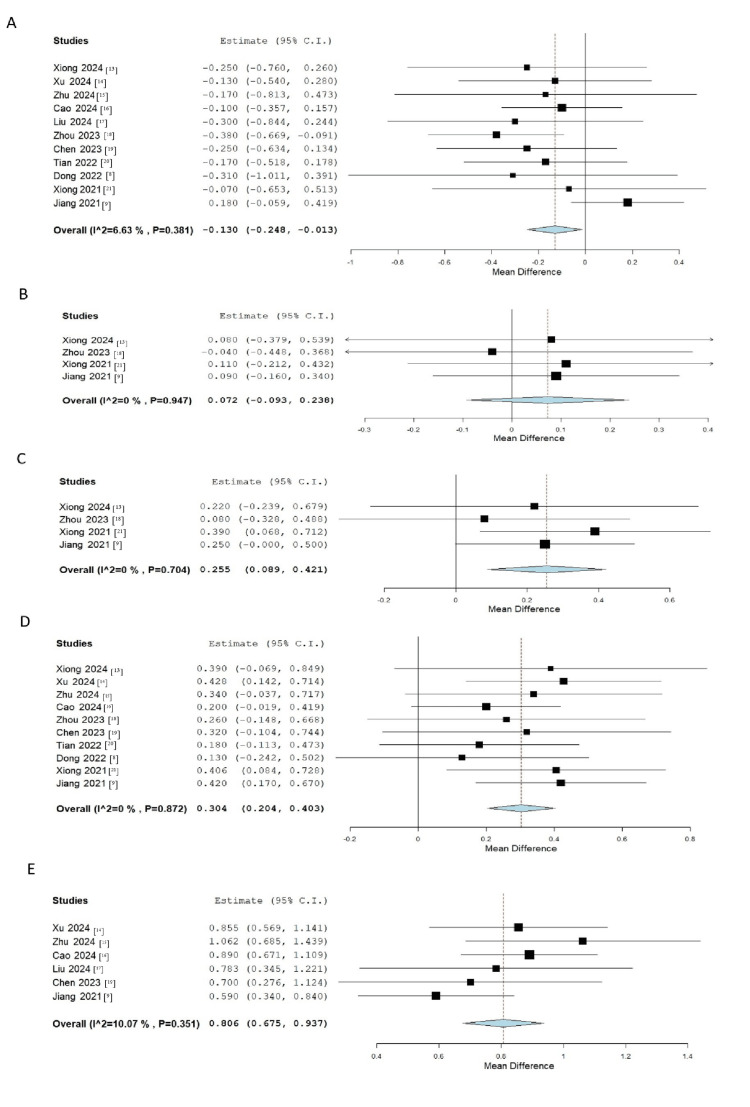
Forest plot illustrating the meta-analysis results comparing the difference in SER between the LLRL and control groups at various follow-up points: (A) baseline, (B) one month, (C) three months, (D) six months, and (E) 12 months LLRL, low-level red light; SER, spherical equivalent refraction [8,9,13-21]

The meta-analysis revealed a statistically significant difference in SER between the LLRL and control groups over time. At baseline, a slight difference favoring the control group was observed, with an estimated value of -0.130 (95% CI: -0.248 to -0.013; p = 0.030) (Figure 3A).

After one month, no statistically significant difference was found between the groups (estimate: 0.072; 95% CI: -0.093 to 0.238; p = 0.493) (Figure 3B). However, at three months, the LLRL group demonstrated a significant improvement in SER compared to the control group, with an estimate of 0.255 (95% CI: 0.089 to 0.421; p = 0.016) (Figure 3C). This trend continued with even greater significance at six months (estimate: 0.304; 95% CI: 0.204 to 0.403; p < 0.001) and 12 months (estimate: 0.806; 95% CI: 0.675 to 0.937; p < 0.001) (Figure 3D, 3E).

**Figure 4:**
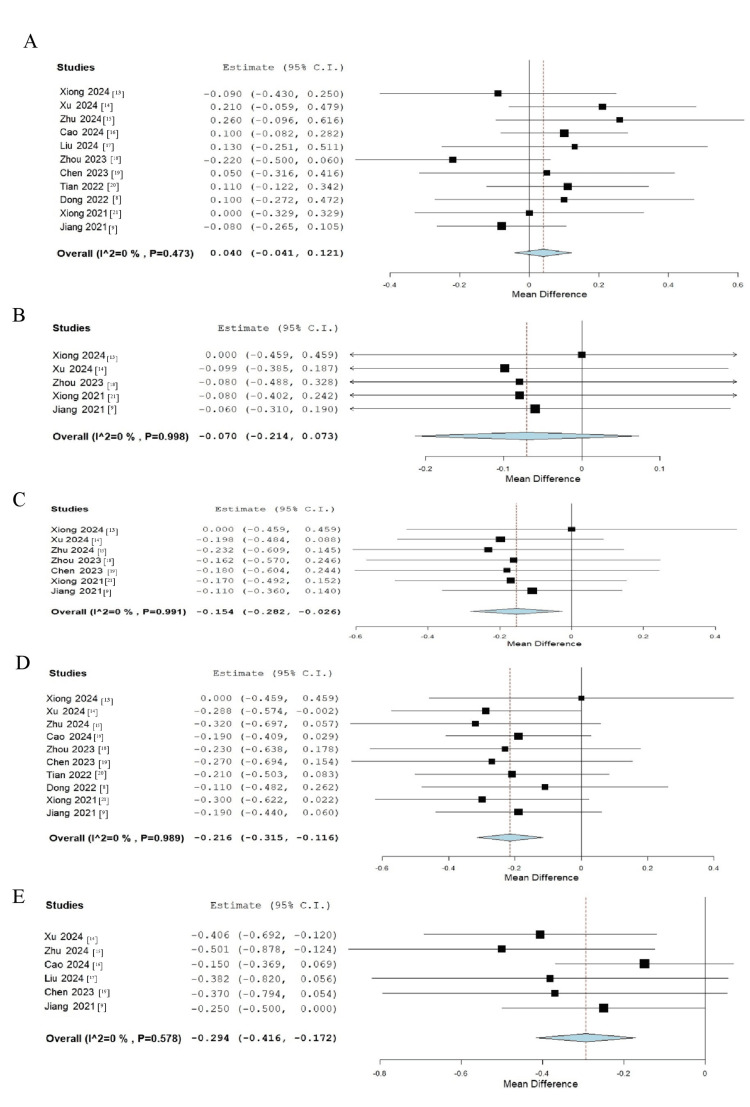
Forest plot illustrating the meta-analysis results comparing the difference in AL between the LLRL and control groups at various follow-ups: (A) baseline, (B) one month, (C) three months, (D) six months, and (E) 12 months AL, axial length; LLRL, low-level red light [8,9,13-21]

Meta-analysis results for AL revealed no significant difference between the groups at baseline, with an estimate of 0.040 (95% CI: -0.041 to 0.121; p = 0.336) (Figure 4A). Similarly, at one month, the difference between the groups remained statistically insignificant (estimate: -0.070; 95% CI: -0.214 to 0.073; p = 0.473) (Figure 4B). However, by three months, a borderline significant reduction in AL was observed in the LLRL group compared to the control group (estimate: -0.154; 95% CI: -0.282 to 0.026; p = 0.062) (Figure 4C). At six months, this reduction reached statistical significance, with an estimate of -0.216 (95% CI: -0.315 to -0.116; p < 0.001) (Figure 4D). By 12 months, the difference in AL had further increased in favor of the LLRL group, with an estimate of -0.294 (95% CI: -0.416 to -0.172; p < 0.001) (Figure 4E).

**Figure 5:**
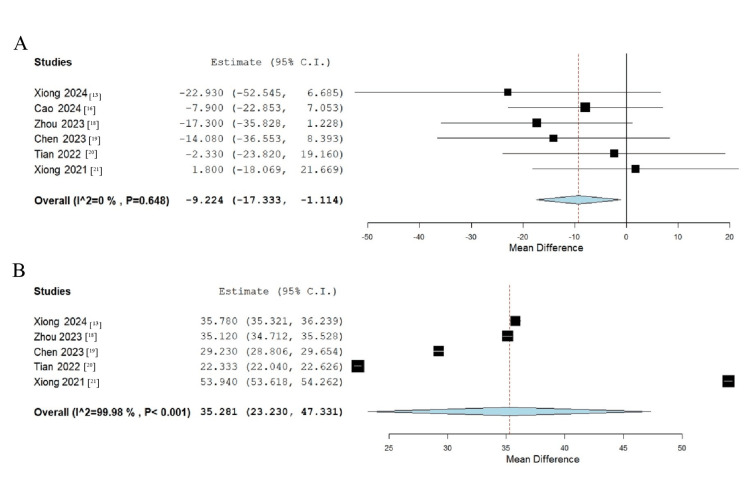
Forest plot illustrating the meta-analysis results comparing SFCT between the LLRL and control groups: (A) baseline and (B) six months LLRL, low-level red light; SFCT, subfoveal choroidal thickness [13,16,18-21]

The meta-analysis comparing SFCT between the LLRL and control groups revealed a significant baseline difference, with the LLRL group showing a decreased SFCT of -9.224 μm compared to the control group (95% CI -17.333 to -1.114; p = 0.026) (Figure 5A). However, after six months of treatment, the LLRL group demonstrated a significant increase in SFCT, with an estimated difference of 35.281 μm (95% CI 23.230 to 47.331; p < 0.001) (Figure 5B).

Discussion

The findings of this systematic review and meta-analysis offer valuable insights into the effectiveness of LLRL therapy in controlling myopia progression in children. Myopia, commonly referred to as “nearsightedness,” is a growing public health concern worldwide, particularly in East Asia, where its prevalence is notably high [22-24]. Emerging evidence suggests that environmental factors, especially those related to light exposure, play a critical role in myopia progression [25-27]. Within this context, LLRL has emerged as a promising intervention aimed at mitigating myopia progression, providing a noninvasive and safe alternative to pharmacological treatments such as atropine eye drops and orthokeratology lenses [20].

Pharmacological interventions have been extensively studied for their effectiveness in controlling myopia. Among them, atropine eye drops remain the most researched and commonly utilized approach. Low-dose atropine has demonstrated substantial efficacy in reducing myopia progression, primarily by inducing choroidal thickening of the eye. However, higher concentrations, such as 1%, are more effective but often come with adverse effects, including photophobia, blurred near vision, and pupil dilation [6,28-30]. Beyond pharmacological options, optical interventions such as orthokeratology lenses and multifocal soft contact lenses have gained popularity in myopia management. These lenses not only correct refractive errors but also help control myopia progression by slowing AL elongation [5,30].

Given the expanding body of research supporting various therapeutic approaches, combining interventions like low-dose atropine with orthokeratology lenses or LLRL therapy may offer optimal myopia control while minimizing side effects [28,31]. The studies included in this review consistently demonstrated that LLRL therapy effectively slowed myopia progression, as indicated by changes in SER and AL, two key markers of myopia progression [32]. Across multiple RCTs, LLRL therapy - delivered through daily exposure to red light at wavelengths of approximately 650 nm for three minutes twice daily - was associated with significantly reduced rates of myopic progression compared to control groups [14,15]. Notably, several studies reported that the LLRL group exhibited not only a deceleration in SER changes but also, in some cases, a stabilization or slight improvement in SER values over the follow-up periods [16,20].

Comparative Effectiveness With Control Groups

The comparison between LLRL treatment groups and control groups across various studies highlighted important differences in myopia progression. Most studies reported consistent myopia progression within control groups, characterized by worsening SER and increased AL over time. For instance, studies by Chen et al. and Xiong et al. [19,21] demonstrated that control groups experienced progressive AL elongation accompanied by corresponding declines in SER, which are hallmark indicators of myopia progression.

In contrast, participants in the LLRL groups exhibited significantly less myopic progression. Jiang et al. [9] reported that the LLRL group experienced a substantially smaller reduction in SER compared to the control group over a 12-month period. Additionally, the increase in AL, a critical factor in myopia progression, was significantly slower in LLRL-treated participants [13]. The impact of LLRL on AL is particularly noteworthy because AL elongation is directly linked to worsening myopia and an elevated risk of pathological conditions, including retinal detachment, myopic maculopathy, and glaucoma [33-35].

The studies included in this review suggest that LLRL therapy effectively inhibits AL elongation, potentially reducing the risk of long-term ocular complications. In control groups across multiple studies, AL progressively increased, with some studies reporting AL elongation exceeding 0.50 mm within one year. In comparison, the LLRL groups exhibited minimal AL increases, with some studies even reporting stabilization [8,16-20]. This reduction in AL elongation is a critical finding, underscoring the potential of LLRL therapy to address a primary driver of myopia progression.

SFCT and Myopia Control

An additional significant finding from the articles included in this review is the impact of LLRL on SFCT. The choroid plays a crucial role in ocular health, particularly in regulating intraocular pressure and supplying nutrients to the outer retina [36,37]. Thinning of the choroid is commonly associated with myopia progression and related complications [38-40].

In this review, LLRL therapy appeared to exert a protective effect on the choroid, as demonstrated by either the stabilization or increase of SFCT in the LLRL group compared to controls. For instance, studies by Xiong et al. and Zhu et al. [13,15] reported a decline in SFCT within control groups, which is consistent with the typical pattern observed in progressive myopia. In contrast, participants receiving LLRL therapy showed either maintenance or a slight increase in SFCT over time.

These findings suggest that LLRL may enhance choroidal health and potentially reduce the risk of myopia-associated degenerative changes. While the exact mechanisms underlying LLRL’s effects on the choroid remain unclear, it has been proposed that red light may positively influence blood flow within choroidal vessels, promoting a healthier ocular environment and thereby slowing myopia progression [25,41,42].

Furthermore, the preservation of choroidal thickness observed in LLRL-treated individuals aligns with previous research indicating that increased outdoor activity and exposure to natural light can decrease the progression rate of myopia. This further supports the role of light exposure in healthy ocular development [43,44].

Power Settings and Treatment Efficacy

Another key observation from the included studies was the potential influence of higher LLRL power settings on treatment efficacy. One study explored various LLRL power levels, ranging from 0.37 mW to 1.20 mW, and found that higher power settings were associated with greater reductions in myopic progression [18]. This finding aligns with previous research suggesting that more intense light exposure may be more effective in stimulating protective ocular mechanisms. However, further research is necessary to confirm the optimal dosing and power settings for LLRL therapy [45].

For instance, in 2024, Zhou et al. tested three different LLRL power levels and found that the 1.20 mW group exhibited the most significant reduction in myopia progression, followed by the 0.60 mW and 0.37 mW groups. These results indicate that while LLRL is generally effective at lower power settings, higher doses may enhance therapeutic outcomes. However, achieving an appropriate balance between treatment efficacy and safety is crucial, as higher-power settings could pose a greater risk of adverse effects. Notably, no significant side effects were reported in the included studies.

Safety and Long-Term Implications

One of the notable strengths of LLRL therapy is its favorable safety profile. None of the included studies reported significant short-term adverse effects, underscoring its noninvasive nature and safety for use in children. Furthermore, the simplicity and ease of administration of LLRL therapy make it an appealing option for managing myopia in pediatric populations [4].

Limitations of the Review

LLRL therapy offers promising potential in combating the escalating myopia epidemic, particularly among younger populations where the incidence continues to rise. While the preliminary results are encouraging, it is essential to acknowledge the limitations and weaknesses of the current evidence. Most studies included in this review had follow-up periods of 12 months or less, leaving the long-term safety and efficacy of LLRL therapy uncertain [14]. Further research with extended follow-up periods is crucial to determine whether the therapeutic benefits of LLRL are sustained over time and to identify any potential delayed side effects.

## Conclusions

This review provides compelling evidence supporting the efficacy of LLRL therapy for myopia control in children. LLRL demonstrated significant reductions in myopia progression, as evidenced by improvements in SER, a slower increase in AL, and stabilization or slight enhancement of SFCT. As a promising, noninvasive alternative to conventional myopia control strategies, LLRL has the potential to significantly impact childhood myopia management. However, further research is needed to determine the optimal dosing, long-term safety, and efficacy of LLRL therapy, particularly in diverse populations beyond East Asia.
